# Supplementary figures and images for: Epigenetic regulation of the respiratory chain by a mitochondrial distress-related redox signal
Source: Front Cell Dev Biol. 2025 Aug 5;13:1608400. doi: 10.3389/fcell.2025.1608400 (PMC12361244; doi:10.3389/fcell.2025.1608400)

Supplementary Figure 2

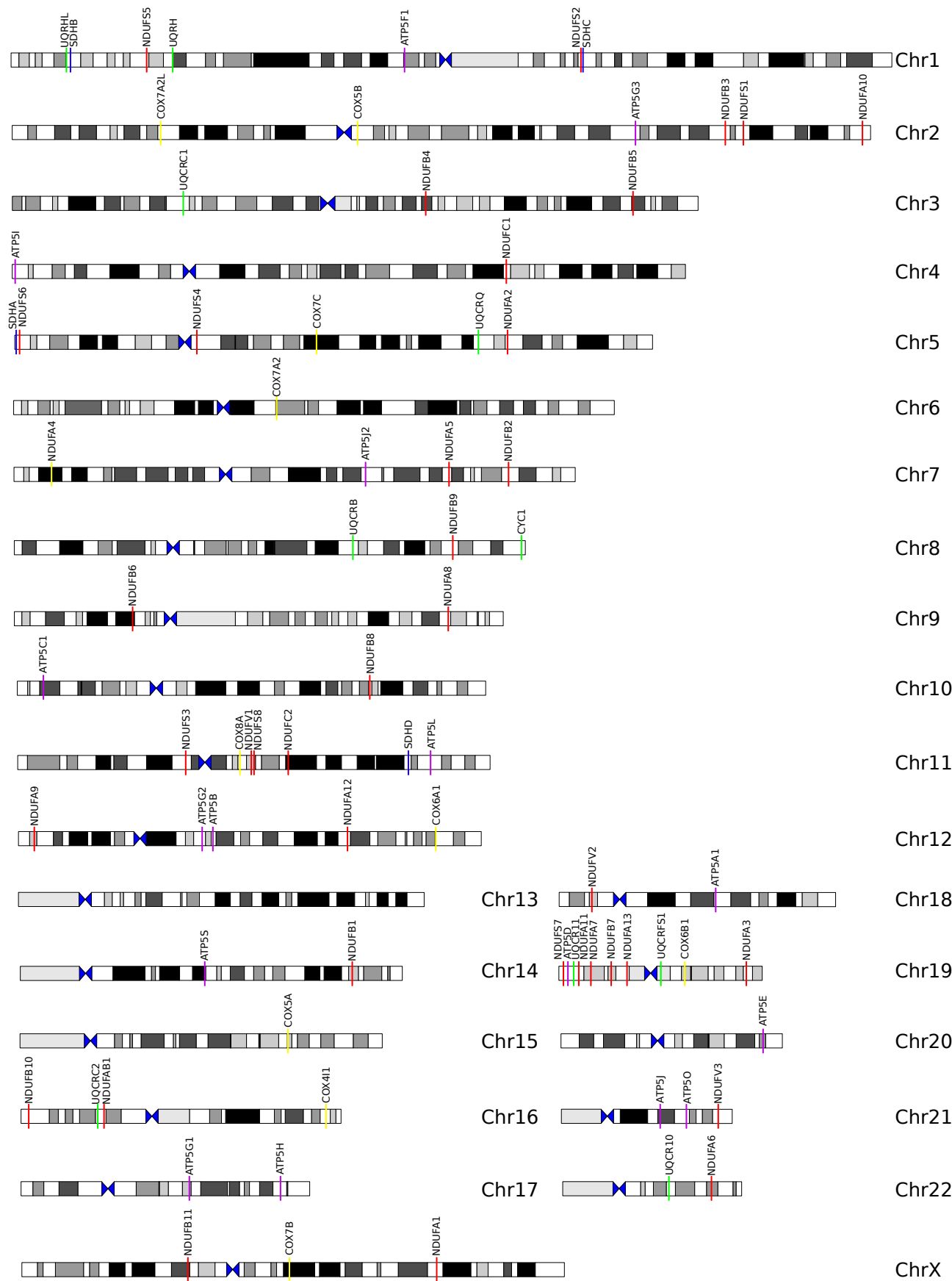

Supplement: Supplementary file 1 [file DataSheet2.pdf]

Supplementary Figure 3

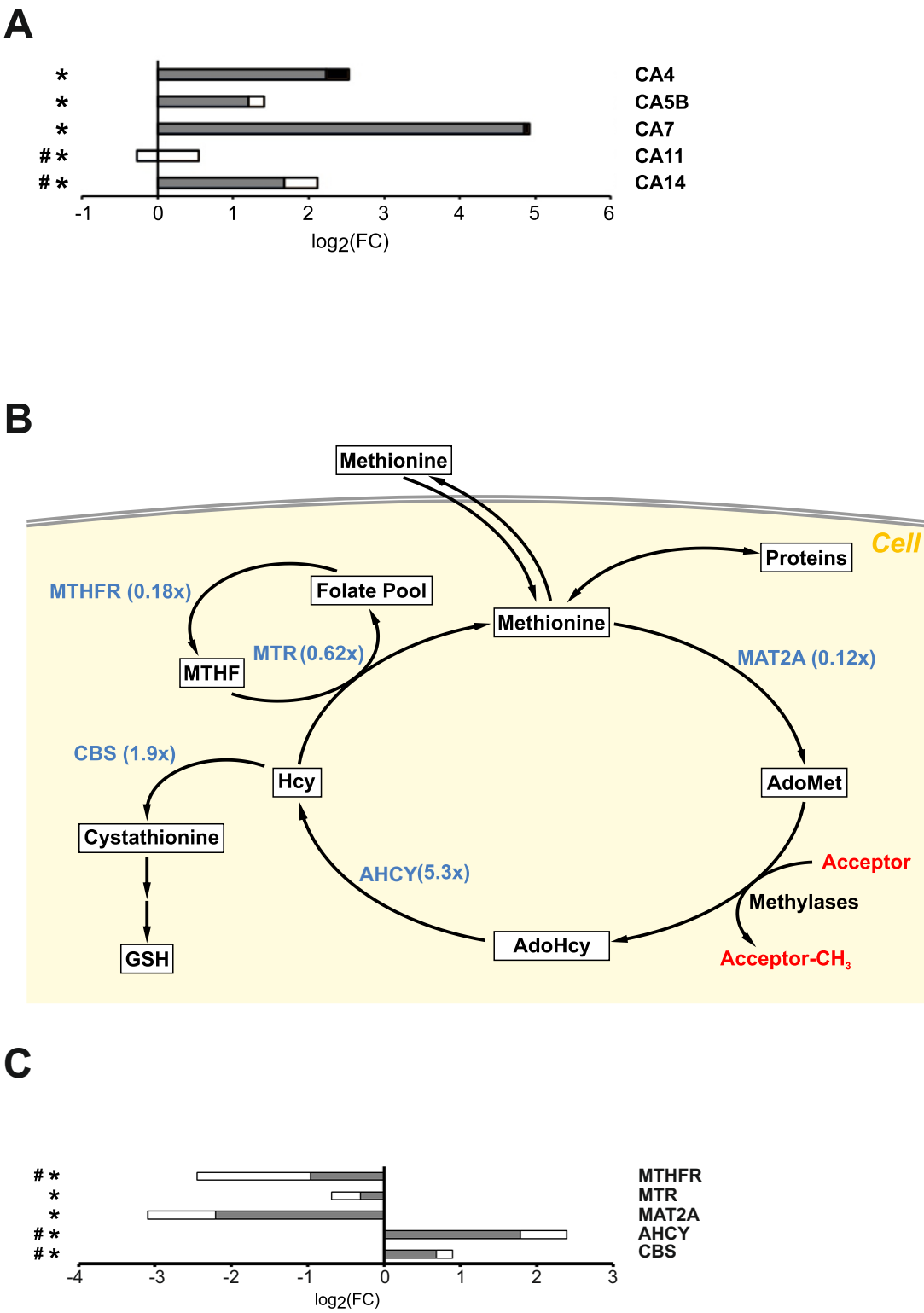

Supplement: Supplementary file 4 [file DataSheet3.pdf]

Supplementary Figure 1

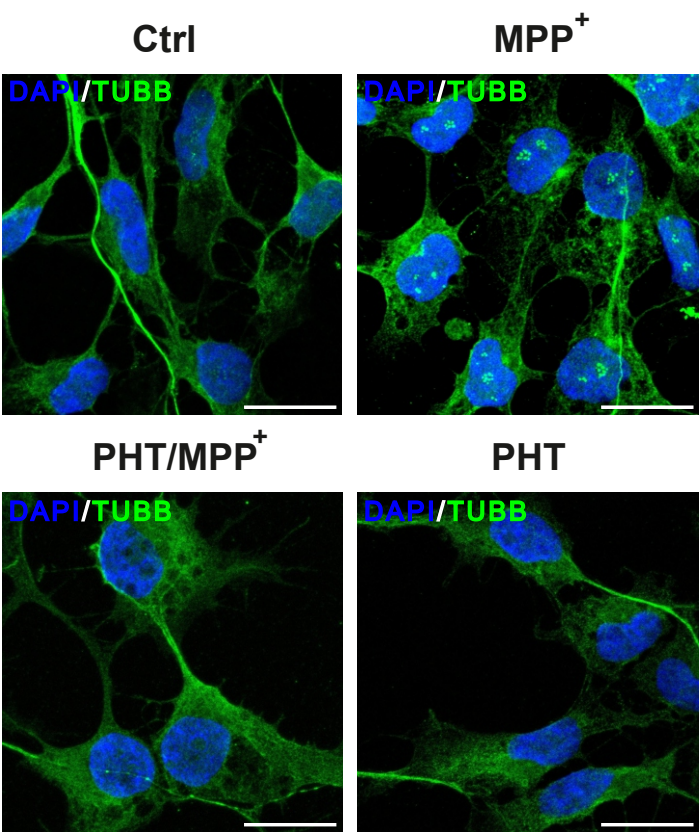

Supplement: Supplementary file 5 [file DataSheet1.pdf]

Supplementary Figure 5

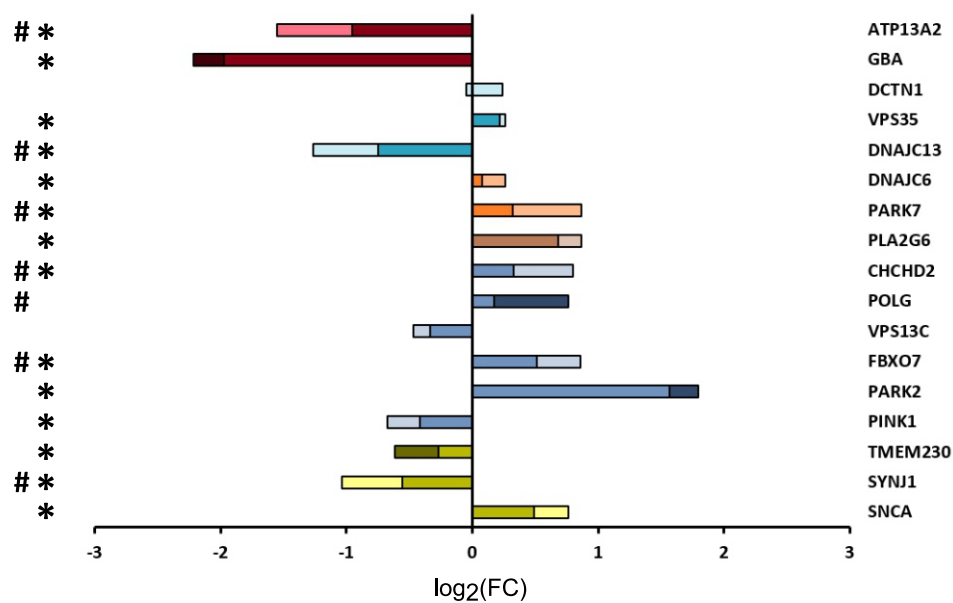

Supplement: Supplementary file 6 [file DataSheet5.pdf]
