# Supplementary material for: Epigenetic regulation of the respiratory chain by a mitochondrial distress-related redox signal
Source: Front Cell Dev Biol. 2025 Aug 5;13:1608400. doi: 10.3389/fcell.2025.1608400 (PMC12361244; doi:10.3389/fcell.2025.1608400)
Supplement: Supplementary file 2 [file DataSheet4.pdf]

Supplementary Figure 4

A

Category	Term	RT	Genes	Count	%	P-Value
GOTERM_MF_DIRECT	metal ion binding	RT		208	16.7	6.3E-16
GOTERM_MF_DIRECT	nucleic acid binding	RT		113	9.1	4.9E-12
GOTERM_BP_DIRECT	transcription, DNA-templated	RT		177	14.2	1.4E-10
GOTERM_BP_DIRECT	regulation of transcription, DNA-templated	RT		142	11.4	3.1E-10
GOTERM_MF_DIRECT	DNA binding	RT		152	12.2	1.3E-9
GOTERM_MF_DIRECT	transcription factor activity, sequence-specific DNA binding	RT		97	7.8	2.0E-8

B

Category	Term	RT	Genes	Count	%	P-Value
GOTERM_BP_DIRECT	SRP-dependent cotranslational protein targeting to membrane	RT		52	5.2	4.1E-38
GOTERM_BP_DIRECT	viral transcription	RT		52	5.2	7.3E-33
GOTERM_BP_DIRECT	translational initiation	RT		55	5.5	8.8E-32
GOTERM_BP_DIRECT	nuclear-transcribed mRNA catabolic process, nonsense-mediated decay	RT		52	5.2	1.2E-31
KEGG_PATHWAY	Ribosome	RT		53	5.3	1.9E-26
GOTERM_CC_DIRECT	ribosome	RT		48	4.8	1.2E-20
GOTERM_MF_DIRECT	structural constituent of ribosome	RT		54	5.4	7.2E-20
GOTERM_BP_DIRECT	rRNA processing	RT		54	5.4	1.4E-19
GOTERM_CC_DIRECT	cytosolic large ribosomal subunit	RT		30	3.0	3.0E-19
GOTERM_CC_DIRECT	cytosolic small ribosomal subunit	RT		24	2.4	3.6E-18
GOTERM_BP_DIRECT	translation	RT		56	5.6	5.4E-18
GOTERM_CC_DIRECT	extracellular matrix	RT		46	4.6	5.7E-13
GOTERM_CC_DIRECT	focal adhesion	RT		58	5.8	3.6E-11
GOTERM_CC_DIRECT	extracellular exosome	RT		203	20.3	2.7E-10
GOTERM_CC_DIRECT	small ribosomal subunit	RT		14	1.4	4.6E-10
GOTERM_CC_DIRECT	extracellular space	RT		87	8.7	2.4E-8
GOTERM_CC_DIRECT	extracellular region	RT		85	8.5	5.6E-7
GOTERM_CC_DIRECT	cell surface	RT		47	4.7	8.8E-7
GOTERM_CC_DIRECT	nucleosome	RT		16	1.6	2.5E-6
GOTERM_BP_DIRECT	cytoplasmic translation	RT		10	1.0	5.9E-6
GOTERM_CC_DIRECT	plasma membrane	RT		215	21.5	6.5E-6

C

Category	Term	RT	Genes	Count	%	P-Value
GOTERM_CC_DIRECT	nucleosome	RT		5	15.6	9.6E-7
GOTERM_BP_DIRECT	nucleosome assembly	RT		5	15.6	7.4E-6

D

Category	Term	RT	Genes	Count	%	P-Value
GOTERM_MF_DIRECT	metal ion binding	RT		78	21.5	3.5E-8
GOTERM_MF_DIRECT	DNA binding	RT		60	16.5	4.6E-6
GOTERM_BP_DIRECT	transcription, DNA-templated	RT		68	18.7	7.0E-6
