# Supplementary material for: Epigenetic regulation of the respiratory chain by a mitochondrial distress-related redox signal
Source: Front Cell Dev Biol. 2025 Aug 5;13:1608400. doi: 10.3389/fcell.2025.1608400 (PMC12361244; doi:10.3389/fcell.2025.1608400)
Supplement: Supplementary file 3 [file DataSheet6.pdf]

RNA Sequencing Data - Ctrl vs. MPP+

Name	Chromosome	Start	End	Strand	log2(FC)	p Value
**Complex I**						
**NDUFV3**	chr21	42879644	42913304	+	1,000651724	6,95841E-14
**NDUFV2**	chr18	9102630	9134345	+	0,925154711	0,011628443
**NDUFV1**	chr11	67606852	67612535	+	1,195623081	6,15531E-25
**NDUFS8**	chr11	68030617	68036644	+	1,600436909	4,59816E-34
**NDUFS7**	chr19	1383527	1395589	+	1,781662381	5,43933E-35
**NDUFS6**	chr5	1801400	1816605	+	1,07253636	1,12843E-19
**NDUFS5**	chr1	39026318	39034636	+	1,028226218	1,44918E-15
**NDUFS4**	chr5	53560633	53683340	+	1,064569411	1,60481E-17
**NDUFS3**	chr11	47565336	47584562	+	0,615413782	3,17149E-06
**NDUFS2**	chr1	161197104	161214395	+	0,240321178	0,017125861
**NDUFS1**	chr2	206114817	206159603	-	-1,087571014	8,48538E-29
**NDUFC2**	chr11	78068304	78080219	-	-2,328646937	1,92301E-62
**NDUFC1**	chr4	139266880	139302551	-	0,932505731	1,1432E-09
**NDUFB9**	chr8	124539103	124568510	+	0,914512784	1,6996E-13
**NDUFB8**	chr10	100523740	100530000	-	0,633374681	2,18838E-09
**NDUFB7**	chr19	14566078	14572062	-	1,31779247	3,54064E-22
**NDUFB6**	chr9	32552999	32573184	-	-0,339182327	0,03580798
**NDUFB5**	chr3	179604690	179627647	+	0,759290491	7,32669E-10
**NDUFB4**	chr3	120596309	120602500	+	1,442170447	1,05961E-33
**NDUFB3**	chr2	201071433	201085750	+	-0,132933734	0,349976497
**NDUFB2**	chr7	140690777	140722790	+	0,828873557	8,66872E-10
**NDUFB10**	chr16	1959508	1961975	+	0,382641065	0,00096539
**NDUFB11**	chrX	47142216	47145504	-	0,883087494	2,49674E-12
**NDUFB1**	chr14	92116122	92121917	-	0,565171122	1,52006E-05
**NDUFAB1**	chr16	23581002	23596356	-	0,773920484	5,67582E-12
**NDUFA9**	chr12	4649095	4694317	+	0,069432398	0,608137682
**NDUFA8**	chr9	122144058	122159819	-	-0,303669864	0,012347438
**NDUFA7**	chr19	8308768	8321396	-	0,601697561	0,305110883
**NDUFA6**	chr22	42085525	42090955	-	1,1521603	6,90022E-19
**NDUFA5**	chr7	123536997	123558255	-	-0,457930271	0,002830609
**NDUFA3**	chr19	54102728	54109257	+	0,726147504	6,63572E-08
**NDUFA2**	chr5	140638740	140647785	-	0,478591334	0,000652437
**NDUFA13**	chr19	19515736	19529054	+	1,639708126	6,9479E-10
**NDUFA12**	chr12	94897055	95003770	-	0,588085514	3,73581E-06
**NDUFA11**	chr19	5891276	5904006	-	0,254130406	0,165734893
**NDUFA10**	chr2	239892450	240025402	-	0,428999434	6,01418E-05
**NDUFA1**	chrX	119871487	119876662	+	1,45009911	1,60562E-29
**Complex II**						
**SDHA**	chr5	218241	256700	+	0,789477181	7,48936E-17
**SDHB**	chr1	17018722	17054170	-	0,846977233	2,16682E-13
**SDHC**	chr1	161314257	161375340	+	-0,207808988	0,043230702
**SDHD**	chr11	112086773	112120013	+	-0,221879516	0,208226991
**Complex III**						
**UQCRQ**	chr5	132866560	132868031	+	0,595604371	3,41215E-05
**UQCRHL**	chr1	15807169	15809348	-	1,545471027	2,05492E-26
**UQCRH**	chr1	46303631	46316776	+	1,616487476	1,39183E-44
**UQCRFS1**	chr19	29205321	29213541	-	0,842371272	5,07076E-13
**UQCRC2**	chr16	21952660	21983660	+	1,486230018	8,3954E-45
**UQCRC1**	chr3	48599002	48610976	-	0,970672007	1,59044E-18
**UQCRB**	chr8	96225920	96235634	-	1,649901065	7,84643E-44
**UQCR11**	chr19	1597172	1605490	-	-0,164270067	0,220978862
**UQCR10**	chr22	29767369	29770413	+	0,700065402	2,58391E-08
**CYC1**	chr8	144095027	144097525	+	1,31090087	2,22024E-25
**Complex IV**						
**NDUFA4**	chr7	10931951	10940256	-	0,815015064	2,24177E-10
**COX8A**	chr11	63974607	63976543	+	1,132122764	2,36414E-20
**COX7B**	chrX	77899438	77907373	+	1,118993762	2,39989E-22
**COX7A2**	chr6	75237675	75250323	-	1,145450174	4,26775E-21
**COX7A2L**	chr2	42333546	42425088	-	1,644333961	7,25172E-43
**COX7C**	chr5	86617904	86620962	+	1,518004082	6,73162E-33
**COX6B1**	chr19	35648223	35658861	+	1,220944589	3,38309E-22
**COX6A1**	chr12	120438090	120440742	+	1,212334933	4,02212E-23
**COX5B**	chr2	97646040	97648383	+	1,127230936	2,55553E-18
**COX5A**	chr15	74919791	74938168	-	1,358322265	1,06817E-33
**COX4I1**	chr16	85798633	85807044	+	1,251369497	4,55285E-28
**Complex V**						
**ATP5S**	chr14	50312326	50335558	+	-1,965499758	3,69727E-38
**ATP5O**	chr21	33903453	33915980	-	0,86146233	4,46704E-11
**ATP5L**	chr11	118401154	118431496	+	1,262904128	4,11264E-25
**ATP5I**	chr4	672436	674338	-	1,689435973	1,45326E-34
**ATP5J**	chr21	25716503	25735673	-	0,620007152	1,06886E-07
**ATP5J2**	chr7	99448475	99466331	-	0,406405051	0,002461289
**ATP5H**	chr17	75038863	75046985	-	0,858614412	2,75877E-12
**ATP5G1**	chr17	48892765	48895871	+	0,216089377	0,177041542
**ATP5G3**	chr2	175176258	175184607	-	1,004688428	9,1339E-18
**ATP5G2**	chr12	53632726	53677408	-	2,089297853	1,3632E-69
**ATP5F1**	chr1	111448864	111462773	+	0,461254178	1,1562E-05
**ATP5E**	chr20	59025467	59032382	-	1,108337342	4,07428E-19
**ATP5D**	chr19	1241746	1244826	+	1,315037983	1,04661E-21
**ATP5C1**	chr10	7788129	7807815	+	0,417528227	0,001695898
**ATP5B**	chr12	56638175	56646068	-	0,531320492	1,99735E-06
**ATP5A1**	chr18	46080248	46104334	-	1,327621713	1,37848E-33
**MT Genes**						
**MT-ND1**	chrM	3307	4262	+	-5,623843853	0
**MT-ND2**	chrM	4470	5511	+	-5,114233332	1,3952E-214
**MT-ND3**	chrM	10059	10404	+	-6,944845422	3,26212E-90
**MT-ND4**	chrM	10760	12137	+	-5,598796665	0
**MT-ND4L**	chrM	10470	10766	+	-5,34407726	0
**MT-ND5**	chrM	12337	14148	+	-5,814954382	0
**MT-ND6**	chrM	14149	14673	-	-7,219035206	1,1591E-120
**MT-CYB**	chrM	14747	15887	+	-5,799655231	1,4499E-252
**MT-CO3**	chrM	9207	9990	+	-6,213297763	0
**MT-CO2**	chrM	7586	8269	+	-5,340564484	4,9362E-223
**MT-CO1**	chrM	5904	7445	+	-6,630401711	0
**MT-ATP6**	chrM	8527	9207	+	-3,900895529	2,9583E-154
**MT-ATP8**	chrM	8366	8572	+	-4,422156957	2,7593E-226
**DNA Methyltransferases**						
**DNMT3B**	chr20	32762385	32809356	+	-1,128259815	1,47607E-16
**DNMT3A**	chr2	25227855	25342590	-	-0,113624583	0,332569307
**DNMT1**	chr19	10133345	10231286	-	-1,078267528	2,09888E-24
**Sirtuins**						
**SIRT1**	chr10	67884669	67918390	+	-0,06325178	0,656703635
**SIRT2**	chr19	38878555	38899862	-	0,632635833	1,50274E-07
**SIRT3**	chr11	215458	236931	-	-0,686644785	4,43979E-05
**SIRT4**	chr12	120302316	120313249	+	0,053555132	0,821799272
**SIRT5**	chr6	13574529	13615158	+	-1,007384444	1,1167E-10
**SIRT6**	chr19	4174109	4182604	-	0,138755206	0,402955464
**SIRT7**	chr17	81911939	81921323	-	0,144520125	0,315459274
**Carbonic Anhydrases**						
**CA14**	chr1	150257159	150265078	+	2,112842884	7,3024E-75
**CA11**	chr19	48637942	48646312	-	0,266719654	0,048921756
**CA7**	chr16	66844379	66854153	+	4,855887877	6,94152E-10
**CA5B**	chrX	15688830	15788409	+	1,407778885	4,56689E-16
**CA4**	chr17	60149936	60170899	+	2,232641653	1,02574E-16
**Methionine cycle enzymes**						
**MTHFR**	chr1	11785723	11806920	-	-2,451003295	1,87071E-89
**MTR**	chr1	236795281	236903981	+	-0,68325319	4,77598E-06
**MAT2A**	chr2	85539165	85545280	+	-3,097251765	4,1038E-149
**AHCY**	chr20	34280268	34311802	-	2,39111071	1,52387E-96
**CBS**	chr21	43053191	43076943	-	0,89895295	2,79E-05
**"Parkinson Genes"**						
**ATP13A2**	chr1	16985958	17011928	-	-1,554913575	7,3519E-32
**GBA**	chr1	155234452	155244699	-	-1,974941215	3,85589E-25
**DCTN1**	chr2	74361154	74392087	-	0,189209264	0,110119834
**VPS35**	chr16	46656132	46689518	-	0,263756795	0,020238975
**DNAJC13**	chr3	132417526	132539032	+	-1,262373039	2,02657E-11
**DNAJC6**	chr1	65248219	65415869	+	0,265059966	0,022281949
**PARK7**	chr1	7954291	7985505	+	0,864634929	6,23472E-12
**PLA2G6**	chr22	38111495	38205690	-	0,870114771	2,90733E-09
**CHCHD2**	chr7	56101569	56106576	-	0,800444458	1,76843E-11
**POLG**	chr15	89305198	89334861	-	0,174742718	0,148830503
**VPS13C**	chr15	61852389	62060473	-	-0,467244805	0,258608092
**FBXO7**	chr22	32474676	32498829	+	0,860138463	4,34001E-18
**PARK2**	chr6	161347420	162727771	-	1,565847499	1,79646E-08
**PINK1**	chr1	20633455	20651511	+	-0,671177356	7,33704E-08
**TMEM230**	chr20	5068232	5113103	-	-0,270204446	0,018861759
**SYNJ1**	chr21	32628759	32728048	-	-1,032595541	1,25072E-07
**SNCA**	chr4	89724099	89838315	-	0,764909603	5,19207E-11

RNA Sequencing Data - MPP+ vs. PHT/MPP+

Name	Chromosome	Start	End	Strand	log2(FC)	p Value
**Complex I**						
**NDUFV3**	chr21	42879644	42913304	+	-0,406024506	0,023087751
**NDUFV2**	chr18	9102630	9134345	+	-0,388672522	0,371124215
**NDUFV1**	chr11	67606852	67612535	+	-0,563498072	0,001410321
**NDUFS8**	chr11	68030617	68036644	+	-0,586979296	0,003958417
**NDUFS7**	chr19	1383527	1395589	+	-0,594667581	0,005038913
**NDUFS6**	chr5	1801400	1816605	+	-0,563299777	0,000750179
**NDUFS5**	chr1	39026318	39034636	+	-0,536956905	0,002618882
**NDUFS4**	chr5	53560633	53683340	+	-0,349982902	0,033075926
**NDUFS3**	chr11	47565336	47584562	+	-0,482011991	0,008629882
**NDUFS2**	chr1	161197104	161214395	+	-0,16400312	0,240844164
**NDUFS1**	chr2	206114817	206159603	-	0,052006614	0,778988503
**NDUFC2**	chr11	78068304	78080219	-	-0,018304604	0,949720011
**NDUFC1**	chr4	139266880	139302551	-	-0,319383664	0,126348334
**NDUFB9**	chr8	124539103	124568510	+	-0,631871682	0,000116307
**NDUFB8**	chr10	100523740	100530000	-	-0,326190997	0,031964751
**NDUFB7**	chr19	14566078	14572062	-	-0,623921786	0,00369744
**NDUFB6**	chr9	32552999	32573184	-	-0,127806897	0,551384237
**NDUFB5**	chr3	179604690	179627647	+	-0,426639792	0,00417604
**NDUFB4**	chr3	120596309	120602500	+	-0,333377101	0,039013114
**NDUFB3**	chr2	201071433	201085750	+	-0,100701495	0,527302969
**NDUFB2**	chr7	140690777	140722790	+	-0,557994496	0,002336706
**NDUFB11**	chrX	47142216	47145504	-	-0,636513117	0,000363931
**NDUFB10**	chr16	1959508	1961975	+	-0,445005107	0,002737431
**NDUFB1**	chr14	92116122	92121917	-	-0,511987023	0,002152043
**NDUFAB1**	chr16	23581002	23596356	-	-0,324875935	0,028720742
**NDUFA9**	chr12	4649095	4694317	+	-0,377950172	0,013650757
**NDUFA8**	chr9	122144058	122159819	-	-0,045733693	0,826162509
**NDUFA7**	chr19	8308768	8321396	-	-0,247885541	0,73829061
**NDUFA6**	chr22	42085525	42090955	-	-0,523599726	0,002995976
**NDUFA5**	chr7	123536997	123558255	-	-0,111345334	0,517124339
**NDUFA3**	chr19	54102728	54109257	+	-0,552908283	0,004350019
**NDUFA2**	chr5	140638740	140647785	-	-0,487319471	0,009397964
**NDUFA13**	chr19	19515736	19529054	+	-0,65420131	0,035924147
**NDUFA12**	chr12	94897055	95003770	-	-0,17752495	0,296378607
**NDUFA11**	chr19	5891276	5904006	-	-0,89450194	0,001182864
**NDUFA10**	chr2	239892450	240025402	-	-0,291968411	0,033795011
**NDUFA1**	chrX	119871487	119876662	+	-0,53877252	0,001111634
**Complex II**						
**SDHA**	chr5	218241	256700	+	-0,119325815	0,405371544
**SDHB**	chr1	17018722	17054170	-	-0,182013856	0,261319058
**SDHC**	chr1	161314257	161375340	+	0,031937583	0,861880581
**SDHD**	chr11	112086773	112120013	+	0,155161128	0,405279492
**Complex III**						
**UQCRQ**	chr5	132866560	132868031	+	-0,524215016	0,005932007
**UQCRHL**	chr1	15807169	15809348	-	-0,435396741	0,017012718
**UQCRH**	chr1	46303631	46316776	+	-0,459164679	0,005506651
**UQCRFS1**	chr19	29205321	29213541	-	-0,397195879	0,010404127
**UQCRC2**	chr16	21952660	21983660	+	-0,434136519	0,001527239
**UQCRC1**	chr3	48599002	48610976	-	-0,443483545	0,007836787
**UQCRB**	chr8	96225920	96235634	-	-0,575611316	8,22167E-05
**UQCR11**	chr19	1597172	1605490	-	-0,255619116	0,151696445
**UQCR10**	chr22	29767369	29770413	+	-0,26683673	0,139553949
**CYC1**	chr8	144095027	144097525	+	-0,570892987	0,003019241
**Complex IV**						
**NDUFA4**	chr7	10931951	10940256	-	-0,465209015	0,005841188
**COX8A**	chr11	63974607	63976543	+	-0,603336039	0,003384294
**COX7B**	chrX	77899438	77907373	+	-0,505582724	0,002397111
**COX7A2**	chr6	75237675	75250323	-	-0,439664912	0,007417757
**COX7A2L**	chr2	42333546	42425088	-	-0,513851773	0,002629389
**COX7C**	chr5	86617904	86620962	+	-0,456683389	0,005628517
**COX6B1**	chr19	35648223	35658861	+	-0,504666372	0,010404127
**COX6A1**	chr12	120438090	120440742	+	-0,419828148	0,016011442
**COX5B**	chr2	97646040	97648383	+	-0,603248025	0,000800313
**COX5A**	chr15	74919791	74938168	-	-0,501795882	0,001031499
**COX4I1**	chr16	85798633	85807044	+	-0,558828758	0,001181167
**Complex V**						
**ATP5S**	chr14	50312326	50335558	+	0,163998354	0,548836472
**ATP5O**	chr21	33903453	33915980	-	-0,509426621	0,002304464
**ATP5L**	chr11	118401154	118431496	+	-0,608687754	0,000366567
**ATP5I**	chr4	672436	674338	-	-0,583051975	0,002844265
**ATP5J**	chr21	25716503	25735673	-	-0,378572058	0,023379366
**ATP5J2**	chr7	99448475	99466331	-	-0,538629778	0,002616587
**ATP5H**	chr17	75038863	75046985	-	-0,474284801	0,001356493
**ATP5G1**	chr17	48892765	48895871	+	-0,543920007	0,007809262
**ATP5G3**	chr2	175176258	175184607	-	-0,559191831	0,000848851
**ATP5G2**	chr12	53632726	53677408	-	-0,639304071	0,000596755
**ATP5F1**	chr1	111448864	111462773	+	-0,478304443	0,000631206
**ATP5E**	chr20	59025467	59032382	-	-0,573831305	0,000579036
**ATP5D**	chr19	1241746	1244826	+	-0,695985029	0,004732985
**ATP5C1**	chr10	7788129	7807815	+	-0,422603933	0,007948493
**ATP5B**	chr12	56638175	56646068	-	-0,539786147	0,000232508
**ATP5A1**	chr18	46080248	46104334	-	-0,573710827	0,000155834
**MT Genes**						
**MT-ND1**	chrM	3307	4262	+	0,143893778	0,609969408
**MT-ND2**	chrM	4470	5511	+	0,006318292	0,986014379
**MT-ND3**	chrM	10059	10404	+	0,514965187	0,091566515
**MT-ND4**	chrM	10760	12137	+	0,042424039	0,880061264
**MT-ND4L**	chrM	10470	10766	+	-0,010007964	0,976156838
**MT-ND5**	chrM	12337	14148	+	0,013514749	0,962853381
**MT-ND6**	chrM	14149	14673	-	0,445754157	0,627735452
**MT-CYB**	chrM	14747	15887	+	0,126971366	0,816881594
**MT-CO3**	chrM	9207	9990	+	0,260830035	0,370336162
**MT-CO2**	chrM	7586	8269	+	0,065697111	0,835687817
**MT-CO1**	chrM	5904	7445	+	-0,067274979	0,790581232
**MT-ATP6**	chrM	8527	9207	+	0,096324576	0,747372851
**MT-ATP8**	chrM	8366	8572	+	-0,104356926	0,729130123
**DNA Methyltransferases**						
**DNMT3B**	chr20	32762385	32809356	+	0,141525692	0,522201951
**DNMT3A**	chr2	25227855	25342590	-	0,211654007	0,135434952
**DNMT1**	chr19	10133345	10231286	-	-0,003781617	0,986596544
**Sirtuins**						
**SIRT1**	chr10	67884669	67918390	+	0,113885216	0,50199389
**SIRT2**	chr19	38878555	38899862	-	-0,264009786	0,119142338
**SIRT3**	chr11	215458	236931	-	0,417727134	0,047647141
**SIRT4**	chr12	120302316	120313249	+	0,558283963	0,021479493
**SIRT5**	chr6	13574529	13615158	+	0,126248996	0,654994872
**SIRT6**	chr19	4174109	4182604	-	-0,147284293	0,58293472
**SIRT7**	chr17	81911939	81921323	-	0,154740747	0,456461358
**Carbonic Anhydrases**						
**CA14**	chr1	150257159	150265078	+	-0,439359638	0,00195258
**CA11**	chr19	48637942	48646312	-	-0,541258739	0,0022342
**CA7**	chr16	66844379	66854153	+	0,070266692	0,896765573
**CA5B**	chrX	15688830	15788409	+	-0,199185356	0,359572425
**CA4**	chr17	60149936	60170899	+	0,29730981	0,310058704
**Methionine cycle enzymes**						
**MTHFR**	chr1	11785723	11806920	-	1,487705797	6,66062E-12
**MTR**	chr1	236795281	236903981	+	0,372629722	0,06873311
**MAT2A**	chr2	85539165	85545280	+	0,893746977	4,37599E-05
**AHCY**	chr20	34280268	34311802	-	-0,602972974	0,000686357
**CBS**	chr21	43053191	43076943	-	-0,212421987	0,460147228
**"Parkinson Genes"**						
**ATP13A2**	chr1	16985958	17011928	-	0,597967166	0,000563457
**GBA**	chr1	155234452	155244699	-	-0,241698372	0,501477416
**DCTN1**	chr2	74361154	74392087	-	-0,238904115	0,078107707
**VPS35**	chr16	46656132	46689518	-	-0,045178098	0,783239853
**DNAJC13**	chr3	132417526	132539032	+	0,514854792	0,032963005
**DNAJC6**	chr1	65248219	65415869	+	-0,187825267	0,242190607
**PARK7**	chr1	7954291	7985505	+	-0,541696294	0,000446078
**PLA2G6**	chr22	38111495	38205690	-	-0,185053719	0,381407755
**CHCHD2**	chr7	56101569	56106576	-	-0,468401656	0,007811784
**POLG**	chr15	89305198	89334861	-	0,587499539	0,000356974
**VPS13C**	chr15	61852389	62060473	-	0,130229128	0,665374415
**FBXO7**	chr22	32474676	32498829	+	-0,344421873	0,013726582
**PARK2**	chr6	161347420	162727771	-	0,230811397	0,459025617
**PINK1**	chr1	20633455	20651511	+	0,25154033	0,1206813
**TMEM230**	chr20	5068232	5113103	-	-0,345331032	0,018764952
**SYNJ1**	chr21	32628759	32728048	-	0,474470367	0,060662712
**SNCA**	chr4	89724099	89838315	-	-0,273684048	0,060310891
